# Psychosocial and Cultural Processes Underlying the Epidemiological Paradox within U.S. Latino Sexual Risk: A Systematic Review

**DOI:** 10.3390/bs13030226

**Published:** 2023-03-05

**Authors:** Patricia Cabral, Miya Chinn, Jasmine Mack, Miari Costarelli, Emma Ross, Ethan Henes, Lily Steck, Alika Jay Ka’imipono Williams, Yoo Bin Lee, Sofia Fretes, Grace Fernandez, Leslie Garcia, Lucia Sato, Yareimy Patrocinio, Disha Shah

**Affiliations:** Psychology Department, Occidental College, 1600 Campus Road, Los Angeles, CA 90041, USA

**Keywords:** parental influence, peer norms, *familismo*, acculturation, Latina/o youth, sexual risk, gender norms, religiosity

## Abstract

According to the epidemiological paradox, less acculturated Latina/o youth display fewer sexual risk behaviors. A systematic review was performed on psychosocial and cultural mechanisms potentially underlying the epidemiological paradox in sexual risk behaviors of U.S. Latina/o youth across acculturation measures (between January 2000 to October 2022). Thirty-five publications (*n* = 35) with forty-eight analyses of underlying mechanisms met the inclusion criteria. Thirty-one results from twenty-three publications found supporting evidence that one of the five factors was an underlying mechanism in the epidemiological paradox (*n* = 13 parenting practices, *n* = 4 peer influences, *n* = 4 *familismo* values, *n* = 4 religiosity, *n* = 6 traditional gender norms) as, generally protective, mediators or moderators in the link between acculturation and sexual risk behaviors. Studies varied in the sexual risk behavior examined and measurement of acculturation, but primarily employed cross-sectional designs and recruited samples through schools. Mechanisms that enhance close ties and unity of the family, such as those of *familismo* values and positive parenting, reduce the likelihood of sexual risk behaviors as Latina/o youth become more acculturated. Future directions are discussed which may provide guidance for risk prevention and intervention.

## 1. Introduction

The United States (U.S.) is seeing unprecedented changes in the diversity of its population, particularly in national origin, ethnicity, and language use of its youth. As the largest non-White ethnic group in the U.S. [1], Latina/os comprise the largest and fastest growing minority group of children in the U.S. with an exponential growth in the last two decades across the country [2]. Disease and disability among such a large population can have major public health consequences such that issues affecting the healthy development of Latina/o children should be of national importance [3]. To support healthy development and outcomes among this population, we must understand both distinct cultural and common social influences on health, both to understand etiological processes and identify where interventions should be targeted.

### 1.1. Sexual Risk Behaviors among Latina/o Youth

Because Latina/o adolescents’ birth rates are the highest of any major racial/ethnic group in the U.S. [4] and their sexually transmitted infection (STI) prevalence is more than twice that of Whites [5,6,7], their sexual risk behaviors have been deemed a major public health concern [8]. Preventing or delaying the initiation of sexual risk behaviors in adolescence, therefore, can have significant public health implications. Research is needed to identify processes that influence the initiation of health risk behaviors in this developmental period, particularly among vulnerable groups, to inform efforts at preventing or changing them. Given disparities and potentially distinct influences on health risk behaviors in racial/ethnic groups, it should be valuable to focus such efforts at least in part on specific groups. Here, we focus on Latina/o adolescents’ sexual risk behaviors. We defined *sexual risk behaviors* as sexual behavior(s) among adolescents that place them at an increased risk for the contraction of STIs, HIV/AIDS, or unintended pregnancy, which can include, but are not limited to, age of sexual initiation, lack of condom use, and multiple partners.

#### 1.1.1. Acculturation

Migration plays an important role in the experience of many Latino families. Processes associated with migration, such as acculturation, are important concepts for understanding Latina/o health and health risk behaviors [9,10]. Acculturation is typically associated with when an individual or a family arrived in the U.S. and occurs as a process of change in cultural patterns that results from continuous firsthand contact between people from different cultures [11,12]. As of 2013, more than half of Latina/o youth have at least one parent who was born outside the U.S. [7]. The acculturation process may contribute to challenges Latino families face including language barriers, depleted social resources, experiences of discrimination, and cultural conflicts.

Various approaches have been used to operationalize acculturation, which is a complex psychological and sociological process. One approach has been to apply psychometric scales to measure the degree of acculturation, often in multiple dimensions [13,14]. Another common approach in health research has been the use of proxy variables including English language proficiency or use, ethnic identity, cultural orientation, and generational status [15,16,17,18]. Whereas ethnic identity refers to the degree to which a person views oneself as a member of a particular ethnic group, cultural orientation refers to a person’s feelings toward and levels of engagement in a culture [19]. Generational status, a categorization term used by the U.S. Census Bureau, refers to an individual or his or her parents’ place of birth. The first-generation refers to those who are foreign born. The second-generation refers to those born in the U.S. but with at least one foreign-born parent. The third- or higher generation includes those born here with two U.S. native parents [20,21].

#### 1.1.2. Epidemiological Paradox

Notably, acculturation to the U.S. has been deemed influential to paradoxical health outcomes among Latina/os [22]. In particular, Latina/os have been found to experience health outcomes equal to, or better, than non-Hispanic Whites despite manifesting a lower socioeconomic status that is usually associated with poorer health [23]. This paradoxical finding is especially salient among less acculturated generations of Latina/os, such as those who have recently migrated to the U.S. As migrant individuals and families acculturate to the U.S. over time and generations, their developmental and health outcomes appear to become less favorable, a phenomenon termed the *Hispanic epidemiological paradox*, or alternatively the *immigrant health paradox* [20].

#### 1.1.3. Epidemiological Paradox in Sexual Risk Behaviors

Evidence from epidemiological paradox research has demonstrated that acculturation processes are associated with paradoxical health outcomes and behaviors [24]. In fact, first-generation Latina/o youth have been found less likely to engage in sexual risk behaviors; that is, they report a later age of first intercourse and higher condom use than their later generation peers [25]. Additionally, those with low English-speaking proficiency have reported higher condom use and fewer sexual partners [26,27]. Alternatively, higher English language use has been associated with higher incidence of STIs and unplanned pregnancies [28,29]. Research has begun to explore explanations of this paradox. In fact, early work exploring the immigrant paradox primarily focused on the biophysiological effects of the phenomenon, largely ignoring the psychosocial factors. In line with the biopsychosocial model of health and wellness [30], we propose that understanding the epidemiological paradox in sexual risk behaviors for Latina/o youth can be aided through a focus on intrapersonal and interpersonal-level psychosocial and cultural processes that have been found to change as acculturation occurs.

#### 1.1.4. Proposed Underlying Mechanisms of the Epidemiological Paradox

Psychosocial and cultural factors may help us understand why we find paradoxical outcomes for health risk behaviors, including sexual risk behaviors. For example, social and cultural characteristics have been found to mediate these associations [31]. Moreover, influences on adolescent health risk behaviors can range from intrapersonal, to interpersonal, to broader contextual factors according to the socioecological model of human development [32]. In fact, according to studies guided by the theory of planned behaviors [33], which emphasizes the role of attitudes, subjective peer norms, and perceived behavioral control in predicting an individual’s intentions to engage in a behavior, psychosocial and cultural factors such as parenting practices and peer associations are important influences on decision-making processes which predict health behaviors [34,35]. We propose a focus on intrapersonal and interpersonal social and cultural features, which are the most proximal level of influences on behaviors and have also been examined in the majority of studies on this topic.

**Social processes.** A large body of work has emphasized the influence of psychosocial processes on health risk behaviors that are found across cultures and contexts. Two domains of psychosocial influences on adolescent health risk behaviors including parenting features (i.e., monitoring, involvement, and communication) and peer influences (i.e., peer norms and modeling). These parental and peer factors may underlie paradoxical findings among Latina/os by both influencing sexual risk behaviors as well as change across acculturation.

*Parental influences*. Parental influence encompasses a wide range of attitudes or actions (e.g., monitoring, communication) that somehow shapes or molds the child’s attitudes or behaviors. Many parenting practices may serve as protective influences against engaging in risk behaviors among youth. When adolescents perceive low levels of key parental behaviors, such as monitoring, they are more likely to engage in various health risk behaviors [36,37,38]. Additionally, parenting practices can also vary with acculturation. In fact, parenting practices, such as permissiveness, change among Latinos with each successive generation in the U.S. with an increase in proportion of permissive parents [39]. Subsequently, parenting influences may explain differences in sexual risk behaviors associated with acculturation.

*Peer influences*. Similarly, to parental influences, peer factors vary according to acculturation and, in turn, influence sexual risk behaviors. Peer influences appear to exert the ability to influence individual behavior among members of a group based on group norms. Specifically, peer norms, or perceiving that peers are engaging in a risk behavior, have been associated with the initiation and engagement of various health risk behaviors [40,41,42]. Moreover, Latina/o adolescents whose families were more recent migrants to the U.S. have been found to be more resistant to peer pressure than those of later generations [43]. Although important, these typical social processes of parental and peer influences do not account for the unique cultural context of Latina/o youth that may contribute to the epidemiological paradox.

**Cultural processes.** One hypothesis posited to explain the epidemiological paradox includes the erosion of protective cultural processes as acculturation occurs. Research suggests that certain features of the Latino culture may enhance resilience, such as familism and religiousness [44]. Specifically, the retention of some traditional cultural values appears to protect adolescents from engaging in health risk behaviors [45]. However, some cultural factors such as traditional gender norms, particularly among males, increase engagement in health risk behaviors such as violence and sexual risk behaviors [46]. It is therefore important that cultural factors from intrapersonal to interpersonal levels of influence be examined as possible underlying mechanisms of the paradox. Familism, religiosity, and traditional gender norms have been the most highly examined cultural factors in this context.

*Familismo*. Strong family traditions anchor the upbringing of many Latina/o children. Furthermore, the cultural value of *familismo*, which places focus on family as a collective unit while emphasizing family bonding, dependence, obligation, and support [47], reduces the likelihood of sexual risk behavior among Latina/o youth [10,48,49]. However, more acculturated families are less likely to hold onto values and characteristics associated with *familismo* [14,27,47,50]. In fact, first-generation Latinos have the highest levels of family cohesion [51].

*Religiosity*. Within the Latino culture, religiosity is a pervasive force, guiding attitudes, behaviors, and even social interactions for many [52,53,54,55]. Religiosity, referring to the quality or state of religious beliefs or practices, may encompass the endorsement of moral values that guide an individual’s behaviors. In fact, religiosity has a protective association with various sexual risk behaviors including delayed sexual debut and fewer sexual partners [56]. Moreover, less acculturated Latina/os use religious coping strategies more frequently than those with higher levels of acculturation [57]. This deterioration of protective religious dimensions among more acculturated Latina/os may account for the epidemiological paradox in sexual risk outcomes observed among Latina/o youth.

*Traditional gender norms*. Traditional gender norms among Latina/o males and females are often attached to sexual values including notions of female virginity, sexual desire, and sexual communication, as well as affect sexual behaviors [58,59]. Specifically, *machismo,* whereby males are expected to adhere to a heightened masculine role and have little control over sexual impulses, has been associated with unprotected sex and more sexual partners among Latino adult males [60]. On the other hand, ascribing to *marianismo*, a traditional aspect of the female gender role that emphasizes virtues such as purity and moral strength, has been associated with reduced sexual activity among females [61]. However, inconsistent condom use has been associated with both of these gender norms among Latina/o youth [58]. Moreover, as acculturation levels increase, Latina/o individuals replace their traditional view and practice of gender roles with those of the mainstream U.S. culture [50,62]. Overall, the influence of cultural factors on sexual behaviors across acculturation processes is complex but may help us better unravel the epidemiological paradox.

### 1.2. Aims

Thus far, no review has examined the influence of both social processes that cross cultural bounds and Latino cultural factors as underlying mechanisms of the epidemiological paradox for sexual risk behaviors observed among U.S. Latina/o youth. Although there is research supporting that psychosocial and cultural factors operate as underlying mechanisms of the paradox, no theories or implications have been reached to illuminate why such factors explain paradoxical findings in this area which could aid in prevention strategies. Additionally, method strategies employed in such studies including the measurements used to examine the epidemiological paradox, such as generational status, language proficiency, and ethnic identification, have not been critically reviewed in this body of research. Moreover, consensus has not been established whether some cultural factors, such as traditional gender norms, lend to resiliency or risk for sexual risk behaviors among Latina/o youth.

Thus, this systematic review will (1) synthesize findings of intrapersonal and interpersonal social (i.e., parental and peer influences) and cultural (i.e., *familismo*, religiosity, and traditional gender norms) factors that have been found to moderate and/or mediate the epidemiological paradox for sexual risk behaviors among U.S. Latina/o youth in that the measure of acculturation will interact significantly with the sociocultural variables to increase or decrease sexual risk outcomes; (2) critique this body of research; (3) propose future directions; and (4) draw implications from this research.

## 2. Method

### 2.1. Search Strategy

The search strategy employed the PRISMA (Preferred Reporting Items for Systematic Reviews and Meta-Analyses) guidelines. A search of Google Scholar, PubMed, PsychINFO, EBSCO, and reference lists of eligible papers was conducted using the following key terms: Latino adolescent sexual risk behaviors, parental influence, peer influence, generation status, acculturation, (English) language use/preference, epidemiological paradox, *familismo*, gender norms, *marianismo*, *machismo*, and religiosity. A sequential process of examining the title, abstract, and main text content of each article was undertaken, with exclusion of articles occurring at each stage.

### 2.2. Eligibility Criteria

Studies were selected for inclusion according to the following criteria: (1) quantitative empirical publications from peer-reviewed journals published between January 2000–October 2022 in English or Spanish; (2) at least one intrapersonal or interpersonal social (i.e., parental and peer influences) and/or cultural factor (i.e., *familismo*, religiosity, and traditional gender norms) was included in the analysis; (3) a measurement of the epidemiological paradox through indicators of acculturation were included in the analysis; and (4) outcomes examined were sexual risk behaviors among U.S. Latino/Hispanic youth. Interrater reliability of Cohen’s Kappa was used to assess agreement for the inclusion of studies among two coders.

## 3. Synthesis of Review

**Figure 1:**
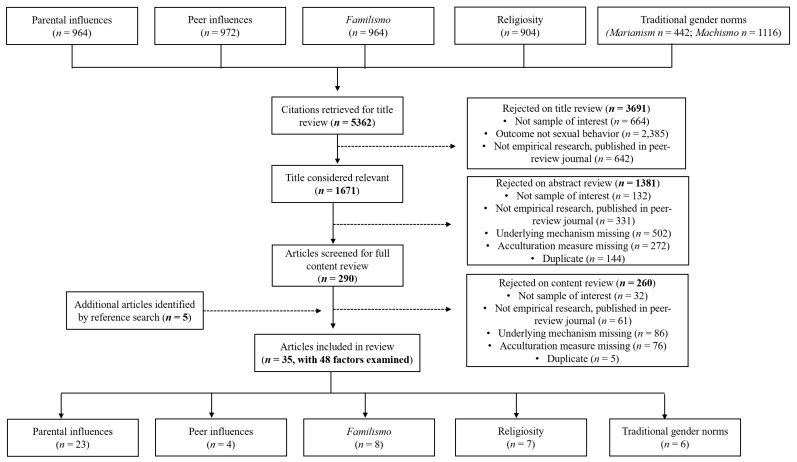
Study selection flow chart. Note: Individual studies that examined multiple factors are counted as separate studies for each factor examined.

Figure 1 delineates the systematic search process. A total of 5362 citations were identified. An additional five studies were identified through reference lists. After screening of the title, abstract, and content for relevance, 5327 were excluded. Of the remaining 35 studies, which included 48 analyses of possible underlying mechanisms, 27 analyses examined social processes (*n* = 23 parental influences; *n* = 4 peer influences) and 21 examined cultural factors (*n* = 8 *familismo*; *n* = 7 religiosity; *n* = 6 traditional gender norms) as moderators or mediators of the epidemiological paradox in sexual risk outcomes among Latina/o youth. All included studies were published in English. Interrater reliability across the factors was high (κ = 0.81–0.97). Identification and coding of included studies was based on two raters for each factor. Each coder extracted information, included in Table 1, and calculated their agreement based on Cohen’s Kappa for extracted study information. About one-fourth of the included studies examined more than one factor (*n* = 13). The majority of the included studies (*n* = 30, over 85%) found significant mediating or moderating effects of sociocultural factors in sexual risk behaviors among Latina/o adolescents. Moreover, most studies that found significant effects examined two types of sexual risk behaviors: (1) use of barrier methods, and (2) initiation of sexual intercourse including intentions. Table 1 details the key characteristics and results of the included publications for each factor. Descriptions regarding the measures of acculturation used within this set of studies are provided in Table 2.

### 3.1. Parenting Influences

Summary. Twenty-three (*n* = 23) studies were retrieved that examined parenting practices and/or behaviors within the epidemiological paradox of Latina/o adolescent sexual risk behaviors (see Table 1). Twelve (*n* = 12) of the studies employed cross-sectional designs [66,68,69,70,71,72,74,75,77,78,79,85] and the other eleven employed longitudinal designs [75,76,77,78,79,80,81,82,83,84,85]. Sexual risk behaviors of interest varied, with four studies examining intentions to have sexual intercourse [64,68,74,75], seventeen focused on sexual activity and condom use [63,65,66,67,69,70,71,72,73,78,79,80,81,82,83,84], one on sexual partners’ risk characteristics [76], and one on knowledge about condom use [77]. Measures of acculturation were also wide-ranging, including generational and nativity status [63,64,67,70,71,72,73,74,76,82,83], acculturation discrepancies between parents and adolescents [65,79,80], language use/proficiency [63,67,69,71,73,77,78,84], and various psychometric scales of acculturation [66,68,74,75,79,80,81].

Results. Eleven studies did not support parenting practices or behaviors as an underlying mechanism between associations with acculturation and sexual risk behaviors among Latina/o youth [63,64,65,69,71,73,76,78,82,83,84,85] in that associations between acculturation, parental influences, and sexual risk behaviors were low and non-significant or parental influences were not found to be significant mediators. However, ten studies found that parenting practices had low to moderate associations with acculturation and sexual behaviors and also partially mediated associations between acculturation and specific sexual outcomes [64,66,67,68,70,71,75,79,80,81]. Additionally, two studies found that acculturation moderated associations between parenting practices and sexual behaviors [72,77].

Specifically, positive parenting practices, such as monitoring and communication, were protective against risky sexual activity in that adolescents with lower acculturation and greater positive parenting practices were approximately half as likely as those of higher acculturation and lower positive parenting behaviors to engage in sexual risk behaviors. In fact, parental acculturation also predicted adolescent sexual risk behaviors and was partially mediated by parenting practices [79]. Moreover, parental influences had an indirect effect on adolescent sexual risk behaviors through dating behaviors, such that greater acculturation among females was associated with a perceived lower maternal approval of dating which was associated with a lower likelihood of being in a relationship, which, in turn, predicted lower intentions to engage in sex in the future [68].

### 3.2. Peer Influences

Summary. Four studies examined peer influences as underlying mechanisms of the epidemiological paradox in Latina/o adolescent sexual risk behaviors. One employed a longitudinal design [64] and all others employed cross-sectional designs [69,75,79]. Outcomes examined included sexual behaviors by three studies [64,69,79] and sexual intentions by one study [70]. One study used language as a proxy of acculturation [69], two used nativity and/or generational status [64,75], and one used psychometric scales to measure acculturation discrepancies between parent and child [79]. Peer influences examined within the selected studies included peer pressure [69], deviant peer affiliations [75], and perceived peer sexual behavior [64,79].

Results. Three of the studies found that peer influences partially mediated associations between acculturation and sexual risk behaviors [64,75,79]. Specifically, perceived peer sexual behavior had a negative and low association to sexual risk behaviors by way of an acculturation gap between parents and adolescent children [79]. Moreover, deviant peer affiliations were a secondary mediation pathway between acculturation and sexual risk behaviors by way of parental influences. In fact, among greater acculturated Latina/o youth, lower paternal acceptance and disclosure to mothers were related to greater deviant peer affiliations, and greater deviant peer affiliations were moderately linked to greater intentions for sex [75].

### 3.3. Familismo

Summary. Eight studies examined values of *familismo* [65,66,68,76,86,87,88]. The studies were evenly split in employing cross-sectional designs [59,60,75,82] and longitudinal designs [65,76,86,88]. Sexual risk behaviors examined varied from intentions to have sexual intercourse [68], sexual activity, condom use and attitudes toward condom use [65,66,86,87,88], to sexual partners’ risk characteristics (i.e., had concurrent partners, used alcohol and/or marijuana at least weekly, and belonged to a gang or was incarcerated during their sexual relationship) [76]. Measures of acculturation were also wide-ranging, including length of time in the U.S. [88], generational and nativity status [76,86], acculturation discrepancies between parents and adolescents [65], language use [86], and various scales of acculturation [66,68,85,87].

Results. Three of the studies did not support *familismo* as an underlying mechanism between associations of acculturation and sexual risk behaviors among Latina/o youth [65,76,85,87] in that associations between acculturation and/or *familismo* or sexual outcomes were not significant. However, four studies found that *familismo* partially mediated associations between acculturation and some sexual behaviors by its own moderate associations with each variable [66,68,86,88]. In fact, *familismo* values were protective against risky sexual activity so that adolescents with higher acculturation and less *familismo* values were almost twice as likely as those of lower acculturation to engage in sexual risk behaviors. Moreover, *familismo* had an indirect effect on sexual risk behaviors through parental and peer influences, such that greater acculturation among males was associated with a lower preference of a romantic partner’s embracement of *familismo*, which, in turn, was associated with greater intentions to engage in sex in the future [68].

### 3.4. Religiosity

Summary. Seven studies examined religiosity as an explanatory mechanism [77,89,90,91,92,93,94], six of which were cross-sectional and one longitudinal [92]. Whereas five of the studies examined sexual activity and condom use [89,90,91,92,94], one examined knowledge about condom use [77], and one examined both voluntary and involuntary sexual activity as an outcome [93]. Two of the studies included religious affiliation along with a measurement of religiosity [77,92] and one examined positive religious coping [91]. Six of the studies examined some form of linguistic acculturation, one additionally included a measure of nativity [89], one included a validated measure of acculturation [91], and two included the length of time in the U.S. [91,92].

Results. Four of the studies found religiosity to be a significant and moderate mediator between acculturation and sexual risk behaviors [89,90,93,94]. Specifically, religiosity appears to fully mediate this association as a protective factor [93] in which less acculturated adolescents with higher religiosity were less likely to engage in various sexual risk behaviors [90]. However, the type of religiosity is associated with whether it is protective or risk-enhancing, such that intrinsic religiosity is protective while extrinsic increases the likelihood of engaging in risk behaviors [94].

### 3.5. Traditional Gender Norms

Summary. Six studies were retrieved that examined traditional gender norm values of *marianismo* and/or *machismo* [58,61,85,86,91,95]. Five were cross-sectional studies [58,61,85,91,95] and one was a retrospective longitudinal study [86]. Five of the studies examined either sexual behaviors and/or condom use, and one study examined attitudes toward condom use and intentions to use condoms in the future [85]. Two studies used validated scales of acculturation [85,91], all other studies used a measure of linguistic acculturation, and one study additionally included measurement of nativity status [86]. Various values of *marianismo* and *machismo* were examined including importance of female virginity or chastity [58,86,91,95], family pillar [91], importance of satisfying sexual needs [58], considering sexual talk disrespectful [95], and gender role orientation [61,85].

Results. All studies demonstrated support for partial mediation by gender role norms of the link between acculturation and sexual risk behaviors. *Marianismo* and *machismo* values were found to have moderate to low associations with acculturation and delayed engagement in sexual behaviors, particularly among females who regard female virginity as important [58,86,91,95]. Specifically, the lower the acculturation level and the more traditional the gender role orientation, the greater a delay in initiating sexual intercourse [61].

## 4. Discussion

A total of 35 publications, with 48 analyses, evaluated 1 or more of the social or cultural factors that were hypothesized to explain the epidemiological paradox in sexual risk behaviors among Latina/o youth. Thirteen of these studies examined more than one of the underlying factors of interest. Longitudinal designs were employed in 17 studies. Twenty-seven results from the thirty-five studies supported one or more of the social and cultural factors as underlying mechanisms of the epidemiological paradox, with low to moderate associations between acculturation measures, underlying mechanisms, and sexual risk behavior outcomes. The underlying mechanisms examined within these studies were generally found to be protective against engaging in sexual risk behaviors, with the exception of negative peer influences.

Yet, 18 analyses from 13 studies reported non-significant findings. Discrepant findings are likely due to the large variation in how acculturation has been measured and under-representation of several factors of interest within empirical studies, particularly of peer influences and traditional gender norms. Consequently, no firm conclusions should yet be drawn about whether some of the hypothesized underlying mechanisms explain the epidemiological paradox in Latina/o youths’ sexual risk behaviors. However, some interpretations and recommendations for future studies can be made from the current literature.

### 4.1. Protective Parental and Familial Explanatory Mechanisms

Of all the underlying mechanisms reviewed here, parental influences and *familismo* values were most often examined. Moreover, twelve studies supported parental influences and four supported *familismo* as underlying mechanisms of the epidemiological paradox within Latina/o youths’ sexual risk behaviors. Their protective roles in preventing sexual risk engagement may emphasize the importance of parents and families among Latina/o adolescents’ everyday life as they acculturate to U.S. norms. For example, Latina/o children are more likely than children in other racial/ethnic groups to eat dinner with their families six or seven nights a week [7]. However, Latino families are often faced with challenges including economic hardship, discrimination, and neighborhood context often characterized by high crime rates and unstable housing. These challenging contextual features along with parent–child acculturation discrepancies can also influence the parenting practices employed by Latino parents and family cohesion [103,104]. Thus, strengthening positive parenting practices and family cohesion may result in positive outcomes among Latina/o adolescents.

Mechanisms that reflect close familial ties are the most clearly supported in the literature to reduce the likelihood of sexual risk behaviors. For example, *familismo* values are expected to reduce the risk for negative behaviors by cementing strong bonds of attachment to the family, ensuring that the family remains a strong source of influence, and fostering conventional ties that discourage Latina/o youth from engaging in a variety of problem behaviors [62,105,106]. Moreover, family support can mitigate environmental influences such as poor neighborhood quality on health risk behaviors [107] by improving adolescent social-emotional competencies [108]. Moreover, *familismo* values may enhance and provide a context for parents to engage in a broader range of positive parenting practices such as monitoring, involvement, and communication. Some studies in this review examined constructs of both parental and *familismo* influences [65,66] within a single latent variable, possibly reflecting the overlapping role that parental and *familismo* mechanisms share. In fact, *familismo* values may ensure that parents continue to play an important role in their child’s behavior well into adolescence.

### 4.2. Growing Support for Additional Underlying Mechanisms

Although research on peer influences, religiosity, and traditional gender norms as underlying mechanisms is relatively small at this point, some preliminary interpretations can be made based on the limited number of studies available. Whereas there is evidence suggesting that they influence the association between acculturation and sexual risk behaviors among Latina/o adolescents, for some of these factors, it is unclear whether they are strictly protective or risk-enhancing.

Cultural factors, aside from *familismo*, that appear to reduce the likelihood of sexual risk behavior engagement include, among others, religiosity. When controlling for education and socioeconomic status, Latinos use religious coping mechanisms more frequently than their non-Latino White counterparts [109]. Given its prominent role in Latino culture overall, it appears probable that religiosity may be influential during difficult life transitions, such as during the immigration process [110]. Studies examining religious affiliation suggest that Latina/o adolescents who identify with Christian denominations feel that religion impacts family relationships; yet adolescents who come from families in which both parents are not present express that religion exerts a negative influence [111]. Religious values are important within traditional Latino culture and, when maintained, are likely to increase conformity, diminish involvement with delinquent peers, and inhibit participation in deviant activities [112,113]. Moreover, religiosity may enhance the protective influence of parents and family cohesion such that parents may use religion to teach values and exert social control within the family context [114]. In fact, recent research suggests that parental monitoring may mediate the relationship between family religiosity and some sexual risk behavior [115]. 

Alternative to parental influences and *familismo* values, peers can exert an influence in greater odds of an adolescent engaging in health risk behaviors including sexual behaviors [42,64,116,117]. This is particularly important during adolescence as peers increase in importance and become as or more influential than parents [118,119]. Specifically, adolescents in general are influenced by perceptions of their friends’ engagement in sexual behaviors and are more likely to engage in these behaviors to feel like they fit in [120]. Yet, adolescents who have conservative sexual attitudes engage less frequently in sexual behaviors, even if they perceive their peers to be engaging in risky sexual behaviors [121,122]. The same patterns of peer and friend impact apply to Latina/o adolescents, but with one noted difference. Latina teens are particularly influenced by cultural norms, which tend to be more conservative regarding sexuality [123]. Therefore, it can be posited that Latina early adolescents are likely to be more conservative and place greater weight on familial ties over friendship, which suggests that for these girls, peer behavior may not be as influential in their decisions to engage in sexual activities [123]. In fact, studies included in this review demonstrated that, when acculturation is taken into consideration, the weight of peer influences, although still significant, is reduced. Together, strong ties and a shared culture that opposes negative behaviors can lead to reduced negative outcomes among immigrants [31].

Gender role socialization within a Latino family context is influenced by the cultural concepts of *marianismo* for females and *machismo* for males [50]. Additionally, some facets of traditional gender norm values such as the importance of female virginity and satisfaction of sexual needs were associated with a sexual risk behavior in a protective or risk-enhancing way depending on the specific sexual behavior examined [58]. However, acculturation sways the influence of traditional gender roles on sexual risk behaviors. Specifically, among less acculturated females, values of *marianismo* may lend to a less assertive role within romantic relationships, which may raise concern about Latinas’ ability to communicate regarding sexuality such as condom use [95,124].

## 5. Methodological Critique and Limitations

This research field is relatively new and challenging. Serious methodological problems were identified in various studies. First, we found an over-representation of cross-sectional designs. Twenty-seven of the selected studies employed cross-sectional designs. However, psychosocial and cultural factors can have early and long-lasting influence on an individual’s behavior prior to the initiation of most risky behaviors [37]. Longitudinal designs should aid our understanding of early social and cultural factors that influence subsequent risk behaviors. Moreover, examining protective and risk-enhancing factors before children enter adolescence can better inform about critical periods in which interventions can be introduced prior to the initiation of most health risk behaviors.

Second, a broad variation of acculturation measurements has been used. Whereas some studies utilized language as an acculturation proxy, others have depended on nativity or generation status, and yet others relied on validated acculturation scales. However, non-uniform use of acculturation measures raises the question about whether different studies are capturing similar aspects of acculturation. Because of their reliance on unidimensional conceptions of acculturation, most studies are limited in that it is not clear whether the epidemiological paradox is due to immigrants’ acquisition of receiving-culture practices, loss of heritage–culture practices, or both [22]. In fact, some researchers argue that language use or preference measures share only small amounts of variance with more comprehensive measures and may actually capture different aspects of acculturation [125]. Alternatively, there is strong empirical evidence that supports the use of multidimensional models of acculturation over unidimensional approaches [126,127]. Some have even suggested that examining the epidemiological paradox through multidimensional constructs of acculturation may help researchers better understand underlying mechanisms of the epidemiological paradox [22]. Moreover, multidimensional constructs of acculturation should be used to examine whether biculturalism (reflecting the adoption of the receiving culture while retaining the heritage culture) [128] is the most adaptive approach to acculturation as it has been linked to better outcomes, especially among young immigrants [129,130].

Third, sample recruitment procedures evidence several limitations. Specifically, most studies relied on recruitment from educational institutions. However, due to the higher high-school drop-out rates of Latina/o in comparison to African American and White youth [131], school-based samples may not be representative of Latina/o youth across the U.S, including those at highest risk for sexual risk behaviors. Additionally, a lack of information among many studies regarding the country of origin for each study sample was notable. This is an important part of the context for understanding the results. However, the majority of studies described their sample with general terms of Hispanic or Latina/o as these populations are often treated as homogeneous. Moreover, among those that included country of origin information for their sample, most were predominantly made up of Latina/os from Mexican origins. Yet, studies have produced different results in adult samples when considering the epidemiological paradox in various health outcomes dependent on the country of origin (e.g., Puerto Rico, Mexico, Cuban) [132]. This may be due to differences in perceived discrimination, reasons for migrating, and context of reception, referring to immigrants’ opportunities in the U.S. For example, Mexicans and Puerto Ricans are more likely to be marginalized, whereas Cubans generally fare better [133]. Unlike Mexicans, many of whom are undocumented and seek “under-the-table” positions [134], and Puerto Ricans, many of whom migrate to the northeast and south to escape poverty [135], many Cubans arrive in the U.S. as political refugees (although some do immigrate to escape poverty). The effects of these differences on health risk behaviors in adolescence is not well known. Multisite studies of acculturation and health outcomes are important because acculturation may take different forms depending on the context to which individuals are acculturating [136]. This approach can also aid in capturing greater diversity in Latino group samples, which should be examined as distinct country of origin groups to determine if underlying mechanisms differ by such groups.

Finally, a lack of emphasis on gender differences was evident among some reviewed studies. Despite well-documented gender differences in sexual risk behaviors as well as possible differences in social and cultural processes, some studies did not provide separate analysis of these associations for males and females or lacked consideration for one gender (i.e., exclusive female sample). For example, girls are often more highly monitored by parents and family members than boys [137,138]. Such differences in underlying mechanisms of the epidemiological paradox may further explain differences in significant findings between gender [68,74,75,88]. Analysis of underlying mechanisms between gender would help us better understand whether there are gender differences in their influence as a protective or risk factor and therefore develop better interventions specific to each gender.

## 6. Future Directions

This review suggests several important future directions in this research field. First, additional studies are required to clarify the role of each social and cultural mechanism within the epidemiological paradox of Latina/o adolescent sexual risk behaviors. Specifically, much of the current literature has focused on adult populations or other health risk behaviors among adolescents, particularly substance use. Future studies should streamline the use of acculturation measures, with a preference for multidimensional constructs of acculturation measured by psychometric scales. Subsequently, with improved methodological use of acculturation measurements and further uniformity on the operationalization of psychosocial and cultural factors, meta-analytic techniques should be employed to calculate the overall effect size of these associations.

Pubertal development plays a pivotal role in an adolescent’s sexual risk behavior engagement [139]. Moreover, how and when parents discuss puberty and development with their children may influence how adolescents subsequently handle decisions of sexual behavior [140]. Such discussions may be themselves influenced by acculturation. Moreover, whether parents even discuss puberty with both boys and girls is questionable [141]. In addition, parents’ timing (i.e., before the initiation of sexual behaviors) and the content of pubertal development discussions with their children are important aspects to sexual communication [140]. Therefore, a better understanding is needed of how acculturation can influence parents’ approach and timing to discussions of pubertal development and sexual behaviors with their children and whether this differs between genders.

Decision-making is also an important aspect of engaging in risky behaviors. For example, the theory of planned behavior [33] posits that sexual behaviors involve cognitive processes by which an individual takes a step in deciding whether or not to act on a behavior. In fact, an adolescent’s intentions have been found to be highly associated with whether they will engage in a behavior or not [142,143]. Moreover, psychosocial and cultural factors appear to exert influence on the decision-making process of adolescents’ sexual behaviors across acculturation status [68,74,75]. However, it is unclear why intentions may differ across acculturation levels and how this may be influenced by social and cultural factors. Future studies should expand our understanding of social-cultural mechanisms within cognitive processes of sexual decision-making. Although some of the studies included in this review examined intentions to engage in sex as an outcome, this should be further expanded to examine the factors that influence differences in these intentions across acculturation.

For many Latino families, migration may be a fluid, unexpected, and even temporary experience. The length and timing of migration for Latinos can differ greatly. It is also important to note that in today’s political climate, deportation back to the country of origin can become a reality for both parents and children. Despite being largely ignored within the forced migration literature, it has been argued that deportation is a form of forced migration that warrants attention [144]. The stress of migration processes and fear of deportation may further influence risky behaviors, as seen among Latina/o adults’ drug use, HIV testing, and HIV prevalence in Mexico [145,146]. These stressors may also influence how parents approach their parenting strategies as well as family cohesion [108]. In fact, parenting practices partially mediated the relation between mothers’ age at arrival and young children’s social development, particularly in the case of mothers who arrived as adults [147]. Future studies should examine the role of, not just acculturation, but the migration process undertaken by Latino families when examining the epidemiological paradox. Because features of the migration are shared across groups migrating from different nations, these associations can possibly be examined among various migrant groups.

This field currently lacks a comprehensive model or theory which incorporates the role of acculturation as well as social and cultural factors to explain sexual behaviors of Latina/o youth. Some studies may use previously established models and theories to guide research examining the epidemiological paradox. However, this phenomenon is unique due to the nuanced influence of acculturation on health outcomes and its interaction with sociocultural processes, as well as its specific application to certain ethnic minority groups. Moreover, it is difficult to understand whether and how each underlying mechanism may interact with one another. Although work in this field is still growing, current findings may help the development of a theoretical model that is aimed at explaining the epidemiological paradox in health risk behaviors. Future research should have, as one aim, to support the development of a comprehensive model that accounts for both social and cultural factors across various levels of contexts within an ecological framework. Such a model would piece together the role of each social and cultural factor as well as their interaction with one another within the epidemiological paradox. Moreover, a multilevel model that accounts for broader contexts may help us understand the role of other factors, from cognitive processes to neighborhood characteristics, within the epidemiological paradox.

## 7. Implications

This review points to several important considerations for interventions aimed at reducing sexual risk behaviors among adolescents as well as informs policymakers and health care providers. These findings imply that behavioral public health interventions to prevent engagement in sexual risk behaviors among both U.S.-born and foreign-born Latina/os may need to attend to multiple social-ecological processes, including family and peers, as well as cultural factors uniquely common within this community. Specifically, the integration of parent and family roles in interventions and an emphasis on their involvement in adolescents’ development and maintenance of cultural values may decrease differences in sexual risk behaviors across acculturation, particularly those at increased risk from high acculturation backgrounds. Such integration of typical cross-cultural parenting practices with Latino culturally specific values may also help adolescents develop strategies to resist peer pressure and negative neighborhood influences to which Latino families are often exposed.

Moreover, awareness of the role of acculturation and sociocultural mechanisms within Latino health and health behaviors may provide a culturally sensitive guideline for health care providers. For example, acknowledging that differences in parenting practices and cultural values differ based on acculturation, as indicated by language use, nativity, or generational status, can enhance patient–provider interpersonal interactions and better address cultural-specific beliefs that may influence health outcomes. A clearer understanding of the epidemiological paradox and the underlying sociocultural mechanisms may also provide awareness among policymakers. Policies focused on sex education in public schools may need to address cultural values prominent in different groups.

## 8. Conclusions

This systematic review is the first, to our knowledge, that has examined the social and cultural underlying mechanisms of the epidemiological paradox in sexual risk behaviors among Latina/o youth. This systematic review, covering research published over 20 years (January 2000–October 2022), examined social and cultural mechanisms that were moderators and/or mediators of the epidemiological paradox of Latina/o adolescents’ sexual risk behaviors. The Latino community comprises complex social and cultural factors at the individual, family, community, and societal levels that are critical when addressing adolescent sexual health needs. Latina/o adolescent sexual health is a growing field of research with multiple challenges that need to be addressed. This emerging research field provides contradictory evidence that does not yet clarify the explanatory mechanisms of the epidemiological paradox. Yet, the growing evidence suggests that social and cultural factors that vary by acculturation levels are nuanced predictors of sexual behaviors among Latina/o youth. As more evidence is accumulated, such findings may provide culturally sensitive and relevant intervention guidelines for reducing the high rates of sexual risk behaviors among this population.

## Figures and Tables

**Table 1:** Summary of studies examining underlying mechanisms of epidemiological paradox findings for sexual risk behaviors among Latino youth.

Author(s)	Explanatory Mechanism	Sexual Behavior(s)	Design and Sample	Acculturation Measure(s)	Results
SOCIAL PROCESSES					
Parenting Influence					
Bámaca-Colbert et al., (2014) [63]	Supportive (maternal) parenting and conflict intensity	Age of vaginal sex initiation.	Prospective longitudinal (2006–2010). *n* = 175 Latina females Time 1 age range = 11–17. Mexican origin	Generational status (1st, 2nd, and 3rd+) and language acculturation by BAS ^a^.	Generational status significantly predicted first sexual intercourse (* HR = 0.63). Language acculturation, supportive parenting and conflict intensity were not significant predictors of sexual intercourse.
Cabral et al., (2017) [64]	Parental monitoring, parental involvement, mother nurturance, father nurturance.	Sex initiation by 10th grade.	Prospective longitudinal study. *n* = 1790 (50.1% girls). Mean age = 15.66 (*SD* = 0.65) at 10th grade. Country of origin not recorded.	Generational status based on parental and adolescent nativity.	Parental monitoring significantly predicted 7th grade vaginal intercourse initiation (OR = 0.20) among Latinas. Among Latinas, third-generation were more than twice as likely to initiate oral sex (OR = 5.03) by 7th grade and vaginal (OR = 2.23) intercourse by 10th grade as first- and second-generation Latinas. No significant interactions between parental influences and generational status were found.
Cano et al., (2016) [65]	Family functioning (cohesion, communications, and involvement).	Inconsistent condom use.	Prospective longitudinal (2010–2011). *n* = 302 (47% females) Mean age = 14.51. Country of origin not provided.	Caregiver–adolescent acculturation discrepancies (measured by the BIQ-S ^b^).	Higher levels of caregiver family functioning had significant effects on inconsistent condom use (β = 0.13). No significant indirect effects were found between acculturation components and reports of condom use through family functioning.
Cordova et al., (2014) [66]	Family function discrepancies (i.e., positive parenting, parental involvement, family cohesion, family communication, parental monitoring of peers, and parent–adolescent communication).	Unprotected sex and early sex initiation	Cross-sectional study design. *n* = 746 (47.9% females) (*Mean* age = 13.9 Country of origin not detailed.	Parent and adolescent acculturation using the BIQ-R ^c^.	Higher family functioning discrepancies were significantly related to engaging in early sex initiation (OR = 1.06) and unprotected sex (OR = 1.05). Larger parent–adolescent Hispanicism discrepancies were significantly related to lower adolescent reports of family functioning (OR = 0.70).
Deutsch and Crockett (2016) [67]	Maternal support; Parental control; Parent–child communication about moral issues, health consequences, and recommendation of birth control (BCR).	Sexual intercourse initiation; recent condom use; recent use of birth control	Prospective longitudinal (Add Health data). *n* = 1944 (51% female) Time 1 *Mean* age = 15.46. Country of origin not detailed.	Generational status (youth and parent nativity) and language acculturation (language spoken at home)	Birth control recommendation (BCR) was positively related to sexual intercourse for second-generation youth (β = 0.48). Among first-generation females higher BCR decreased the probability of sexual intercourse 1 year later (OR = 0.22), whereas among second-generation girls, higher BCR increased the probability of subsequent sexual intercourse (OR = 0.63). For second-generation youth only, maternal BCR was significantly associated with increased condom use (β = 0.70). Higher levels of BCR were related to a higher probability of using condoms for second-generation girls and lower probabilities of using condoms for third-generation girls (OR = 0.29).
Guilamo-Ramos et al., (2009b) [68]	Maternal disapproval of dating.	Sexual intercourse intentions	Cross-sectional design. *n* = 400 (54.5% females) *Mean* age = 13.27) Country of origin not detailed.	Mother acculturation: SASH ^d^. Adolescent acculturation: SASH-Y ^e^.	Females: Higher acculturation was associated with a tendency to perceive their mothers as being less approving of dating (β = – 0.18) which was associated with a lower probability of being in a romantic relationship (β = 0.12), which, in turn, predicted lower intentions to have sex (β = 0.33). Higher levels of acculturation also had risk-inducing effects on sexual intentions over and above these mechanisms (β = 0.18). Males: Mother’s acculturation significantly predicted her approval of dating (β = – 0.03), which, in turn, predicted male adolescent’s perception of mother’s approval of dating (β = 0.17). This, in turn, predicted intentions to engage in sex (β = 0.38)
Guzmán and Stritto (2012) [69]	Parental communication of adolescent sexual behavior.	Sexual behaviors (i.e., kissing, making out with clothes on and with clothes off, digital stimulation, oral sex, vaginal sex, and anal sex).	Cross-sectional design. *n* = 217 Latina females *Mean* age = 14.6 (range 13.8 to 15.9 years) Country of origin not detailed.	Language spoken at home by adults.	Both acculturation and parental communication were unrelated to sexual behaviors reported.
Hussey et al., (2007) [70]	Parental presence frequency at home	Sexual intercourse, condom use, number of sexual partners, engaging in sex while under the influence of alcohol or drugs, engaging in sex for drugs or money.	Prospective cohort study (Add Health data) *n* = 3912 (49% females). Asian and Latino (*n* = 2551) adolescents. *Mean* age = 16.1; Country of origin not detailed.	Immigrant status (1st, 2nd, 3rd+ generation).	Compared to foreign-born youth, U.S.-born Asian and Latino adolescents were more likely to engage in sex risk behaviors (2nd gen. *RRR = 2.08; 3rd gen. RRR = 2.02). When accounting for family and residential characteristics, parental presence associated with second-generation immigrant status partly accounted for this finding (RRR = 1.93).
Jimenez, Potts, and Jimenez (2002) [71]	Attitudes toward sexuality and reasons for waiting to have sex.	Contraceptive use, ever had sex, ever been pregnant, and method of contraception used during first and last sexual experiences	Cross-sectional study. *n* = 290 female Latinas *M* age = 15.39. Country of origin not detailed.	Acculturation based on nativity (born in vs. outside the U.S.) and language spoken in the home.	Those born in the US were more likely than immigrants to be sexually active (OR = 3.33). After controlling for acculturation, respondents’ belief that their parents would want them to use protection no longer predicted sexual activity or contraceptive use.
Karoly et al., (2015) [72]	Parent monitoring	Frequency of intercourse and condom use	Cross-sectional design. *n* = 323 (73% male) *Mean* age = 16.07 Country of origin not detailed.	Generational status by parent nativity (first vs. higher generation)	Greater monitoring of location was associated with less risky sexual behavior, but only for youth second-generation and above (β = – 1.05).
Killoren and Deutsch (2014) [73]	Parental monitoring, support, and strictness	Sex with a stranger, 4 or more sexual partners, sexual onset before age 15, common use of contraception that does not protect against STI or pregnancy.	Prospective longitudinal (National Longitudinal Study of Youth). *n* = 1899 Latino youth (49% female) *M* age = 14.29 Country of origin not detailed.	Language spoken in the home and nativity (e.g., citizenship status).	Adolescent males (β = −0.15) born in the U.S. (β = 0.16) had higher sexual risk at Time 2. Adolescents who reported having a strict mother had lower sexual risk at Time 2 (β = – 0.14). However, adolescents who reported a strict father had higher sexual risk at Time 2 (β =.20). Lower paternal monitoring at Time 2 predicted higher sexual risk at Time 3 (β = – 0.03). Nativity status and acculturation were not significant moderators of the relationships between any of the parenting variables and sexual risk at either time point.
Killoren, Updegraff, and Christopher (2011) [74]	Parent–adolescent relationship characteristics (acceptance and time spent with each parent)	Intentions to engage in sexual intercourse	Cross-sectional design. *n* = 246 (50% female) *M* age = 15.70 Mexican-origin adolescents	Cultural orientations using the ARSMA-II ^f^	For males, under conditions of high maternal acceptance, higher Anglo orientations and ( β = 0.15) higher Mexican orientations (β = 0.17) were related to greater sexual intentions. Interactions between cultural orientations, parental relationship characteristics, and sexual intentions were not significant among females.
Killoren, Updegraff, Christopher, and Umaña-Taylor (2011) [75]	Mother– and father–adolescent relationship qualities (i.e., acceptance and disclosure).	Adolescents’ sexual intentions	Cross-sectional design. *n* = 246 (50% female) *Mean* age = 15.7 Mexican-origin youths born in the U.S. and Mexico.	Nativity (born in the U.S. or Mexico), Anglo-Mexican orientation, and generational status (second, third).	For males reporting high maternal acceptance, there was a positive relation between Mexican orientation and sexual intentions, t (207) = 1.92. There was a negative relationship between maternal acceptance and sexual intentions when males reported less orientation to Anglo culture, t (120) = −2.54. These associations did not differ as a function of U.S. youths’ generational status.
Minnis et al., (2010) [76]	Parental monitoring	Sexual partner’s risk characteristics: (1) had concurrent partners; (2) used alcohol/marijuana at least weekly; and (3) belonged to a gang or incarcerated during sexual relationship.	Prospective longitudinal cohort study *n* = 411 (55.4% females) *M* age = 16.6 Country of origin not detailed.	Immigration generation: 4-levels (recent immigrant; 1.5 generation; second generation; and third generation).	Parental monitoring did not mediate the relationship between immigrant generation and either of the two partner risks for which significant differences by generation were found (i.e., partner used alcohol/marijuana frequently and partner gang affiliated/incarcerated). Nonetheless, parental monitoring maintained an independent relationship with partner risk: stronger parental monitoring was associated with decreased odds of choosing higher-risk partners (OR = 0.07).
Nadeem, Romo, and Sigman (2006) [77]	Maternal implicit and explicit communication about contraceptives.	Conceptual knowledge about condom use.	Cross-sectional study. *n* = 45 unmarried pregnant Latina female adolescents *M* age = 16.5) and their mothers. Country of origin not detailed.	Language (Spanish vs. English).	Among Spanish-speaking dyads only, maternal explicit messages were associated with adolescents using more explicit terminology in describing their knowledge (β = 0.55).
Pasch et al., (2006) [78]	Parent–adolescent conflict	Sexual experience (ranging from kissing to sexual intercourse).	Cross-sectional study. *N* = 146 (45% females). *M* = 14.0 Mexican-American families.	Parent and adolescent language acculturation (measured by the SASH ^d^).	When mother and adolescent were both high in acculturation (M = 3.56), adolescents reported higher degrees of sexual experience than when the mother was low and adolescent high in acculturation (M = 2.24; F = 4.05). Yet, mother–adolescent acculturation group was not associated with mother–adolescent conflict and could not be considered a mediator.
Prado et al., (2010) [79]	Social support for parents; parental stressors; and family functioning (parental involvement, positive parenting, family communication, and parent–adolescent communication).	Early sex initiation	Cross-sectional design. *n* = 586 (44.7% female) *Mean* age = 13.6 Country of origin not detailed.	Parents’ U.S. orientation and parent–adolescent U.S. orientation gap (measured by the BIQ-R ^c^).	Parent’s U.S. orientation and early sex initiation were indirectly related through parental stressors, social support for parents, and family functioning (point estimate, β = −0.004). Parent–adolescent U.S. orientation gap and early sex initiation were significantly related through family functioning (point estimate, β = 0.008).
Schwartz et al., (2012) [80]	Parent–adolescent communication	Engaged in oral, vaginal, and anal sex; unprotected oral, anal, or vaginal sex; and number of sexual partners.	Prospective longitudinal design. *n* = 302 (53% boys). *M* age = 14.51, Country of origin not detailed.	Parent–adolescent acculturation gaps (Hispanic and American practices and identifications). Perceived negative context of reception (of one’s ethnic group).	Differential American practices, differential ethnic identity, and both adolescent and parent reports of negative context of reception each indirectly predicted number of sexual partners (β = 1.08, 1.18, 1.24, 0.73, acculturation variables, respectively) and number of oral sex partners (β = 1.06, 1.28, 1.17, 0.62, acculturation variables, respectively) through parent–adolescent communication.
Schwartz et al., (2013) [81]	Family functioning (i.e., parental involvement, positive parenting, and parent-adolescent communication).	Sexual activity and unprotected sex.	Prospective longitudinal design. *n* = 266 (51.9% females) *M* age = 13.4 Country of origin not detailed.	Americanism and Hispanicism (measured by the BIQ ^g^).	Assimilated adolescents reported the poorest family functioning, but adolescent assimilation negatively predicted adolescent sexual activity and unprotected sex indirectly through family functioning (β = −0.07). In families where adolescents perceived family functioning to be more positive than parents did, adolescents appeared to be at greatest risk for sexual activity (β = 0.27) and unprotected sex (β = 0.27). However, acculturation did not predict this discrepancy in reports of family functioning.
Trejos-Castillo and Vazsonyi (2009) [82]	Maternal communication (general and sex); monitoring; and support.	Sex debut year, contraceptive use at first sex and type, STD contraction, multiple sex partners.	Prospective longitudinal study (Add Health data). *N* = 2016 (50.75% females) *M* age = 15.43. Country of origin not detailed.	Immigration status (first and second) and primary language at home.	Neither immigration status nor acculturation moderated the link between maternal parenting constructs and risky sexual behaviors.
Tschann et al., (2002) [83]	Interparental conflict (reported by parent and adolescent)	Sexual activity (ranging from kissing on the lips to sexual intercourse).	Prospective longitudinal study. *N* = 151 (54% boys) *M* = 13:6 Mexican-American origins.	Generational status (first–fourth).	Adolescents’ risk behaviors were linked to several dimensions of parental conflict, when no other variables were taken into account. Language acculturation did not moderate this link.
Upchurch et al., (2001) [84]	Parent–youth relationship (i.e., (socioemotional support, parental control).	Time to first sexual intercourse.	Retrospective longitudinal study. *n* = 497 (50% were between ages 14 – 15; 48% females). Primarily of Mexican origin.	Language of interview (Spanish vs. English).	Compared with females interviewed in Spanish, the risk is 2.6 times as high for females interviewed in English, followed by males interviewed in Spanish (HR = 3.26), with males interviewed in English (HR = 4.49) having the highest risk. Moreover, the hazard ratio is smaller for boys and girls interviewed in English (HR = 1.73), although still significant. Adolescents who report higher levels of parental control have significantly higher risks of first sex (HR = 1.07). When both language of interview and parenting characteristics were included the effect of parental control is reduced to only marginal significance (HR = 1.06, *p* < 0.10).
Velazquez et al., (2017) [85]	Mother–adolescent discussion about sexual topics.	Condom-use attitudes and intentions to use condoms.	Cross-sectional study. *n* = 128 (56.3% female) *Mean age* = 15.30 years, SD = 1.61 Country of origin not detailed.	ARSMA-II ^f^	Acculturation was negatively related to the number of sexual topics discussed between mothers and their adolescents (β =–0.33, *p* = 0.001). Yet, mother–adolescent discussions about sexual topics did not predict condom-use attitudes (*p* = 0.27), or adolescents’ intention to use a condom, *p* =.57.
Peer Influence					
Cabral et al., (2017) [64]	Friendship quality, neighborhood social ties, and peer norms of sex initiation.	Early sex initiation (by 10th grade).	Prospective longitudinal study. *n* = 1790 (50.1% girls) *Mean* age = 15.66 (*SD* = 0.65) at 10th grade. Country of origin not detailed.	Generational status (parental and adolescent nativity).	Third-generation Latinas were more likely than first-generation (OR = 3.50) and second-generation (OR = 2.54) Latinas to initiate vaginal intercourse when at least one friend was perceived to have initiated vaginal intercourse. Third-generation Latinas were more likely than first-generation (OR = 4.96) Latinas to initiate oral sex when at least one friend was perceived to have initiated sexual intercourse. In addition, third-generation Latinas were more likely than first-generation (OR = 4.32) Latinas to initiate oral intercourse by 7th grade when they had a higher number of neighborhood acquaintances.
Guzmán and Stritto (2012) [69]	Normative peer pressure	Sexual behaviors (i.e., kissing, making out with clothes on and with clothes off, digital stimulation, oral sex, vaginal sex, and anal sex).	Cross-sectional design. *n* = 217 Latina girls *M* age = 14.6 Country of origin not detailed.	Language spoken at home.	No significant differences were found in sexual behaviors for normative peer pressure (*χ*^2^ = 3.3, *p* = 0.20) and acculturation (*χ*^2^ = 2.0, *p* = 0.36).
Killoren, Updegraff, Christopher, and Umaña-Taylor (2011) [75]	Deviant peer affiliations.	Adolescents’ sexual intentions	Cross-sectional design. *n* = 246 (50% female) *M* age = 15.7). Mexican origin (U.S. and Mexico born)	Nativity (born in the U.S. or Mexico) and generational status.	Deviant peer affiliations significantly mediated the relations between paternal acceptance and sexual intentions and between disclosure to mothers and sexual intentions for U.S.-born youths (β = 0.46) but not for Mexico-born youths (β = 13). Generational status was negatively related to sexual intentions, but the patterns among parent–adolescent relationship qualities, deviant peer affiliations, and youths’ sexual intentions were identical to those between nativity status (model fit χ^2^(13) = 26.41; CFI = 0.90; RMSEA = 0.09).
Prado et al., (2010) [79]	Perceived peer sexual behavior.	Early sex initiation	Cross-sectional design. *n* = 586 (44.7% female) *M* age = 13.6 Country origin not detailed.	Parents’ U.S. orientation and parent–adolescent U.S. orientation gap (measured by the BIQ-R ^c^).	The parent–adolescent acculturation gap is indirectly related to early sex initiation through perceived peer sexual behavior (β = 0.008). Additionally, parent’s U.S. orientation is associated with adolescent sex initiation through perceived peer sexual behavior (β = −0.004). These associations did not differ between nativity status (Δχ^2^(17) = 21.14, *p* = 0.22).
CULTURAL PROCESSES					
Familismo					
Cano et al., (2016) [65]	Family functioning.	Inconsistent condom use.	Prospective longitudinal design. *n* = 302 (47% females) *M* age = 14.51 Country of origin not detailed.	Caregiver–adolescent acculturation discrepancies (i.e., cultural practices, values, and identities).	Higher levels of caregiver family functioning had significant effects on inconsistent condom use (β = 0.13). However, no statistically significant indirect effects were found between acculturation components and reports of condom use through family functioning.
Cordova et al., (2014) [66]	Family functioning.	Unprotected sex and early sex initiation.	Cross-sectional study. *n* = 746 (47.9% females) *M* age = 13.9 Country of origin not detailed.	Parent and adolescent acculturation using the BIQ-R ^c^.	Findings indicate that higher family functioning discrepancies were significantly related to a higher likelihood of youth engaging early sex initiation (OR = 1.06) and unprotected sex (OR = 1.05). Larger parent–adolescent Hispanicism discrepancies were significantly related to lower adolescent reports of family functioning (OR = 0.70). These findings did not vary by gender for sexual behavior outcomes.
Espinosa-Hernández et al. (2013) [86]	Familism.	Making out, receiving and performing oral sex, and vaginal sex.	Retrospective longitudinal design. *n* = 153 females *Mean* age = 16.3 Mexican origins.	Nativity (born in vs. outside the U.S.) and language use (BAS ^a^).	Mexico-born female adolescents had greater odds of engaging in making out (HR = 1.83) and vaginal sex (HR = 2.07) at earlier ages than female adolescents who were born in the U.S. Females who scored higher on familism were less likely to have made out (HR = 0.93) and performed (HR = 0.93) or received oral sex (HR = 0.94) than female adolescents who scored lower on familism; but did not predict vaginal sex. Moreover, nativity remained a significant predictor of making out (HR = 1.94) and vaginal sex (HR = 1.89).
Guilamo-Ramos et al., (2009a) [68]	*Familismo* and mother’s expectations of *familismo*.	Sexual initiation; condom use at last sex; sexual activity in the past year; number of sex partners; and pregnancy history.	Cross-sectional design. *n* = 702 (47.83% males) *M* age = 13.3 Country of origin not detailed.	Mother acculturation (SASH ^d^) and adolescent acculturation (SASH-Y ^e^).	Adolescents’ acculturation was not associated with sexual risk behavior. Correlations between adolescent *familismo* and adolescent acculturation were low (r = 0.01–0.16), suggesting that acculturation was not associated with adolescents’ embracement of *familismo*.
Guilamo-Ramos et al., (2009b) [87]	*Familismo*.	Intentions to have sexual Intercourse	Cross-sectional design. *n* = 400 (54.5% females) *M* age = 13.27 Country of origin not detailed.	Mother acculturation (SASH ^d^) and adolescent acculturation (SASH-Y ^e^).	Females: Adolescent acculturation significantly predicted their intentions to have sex in the near future (β = 0.18). Although adolescent *familismo* predicted their preference for a romantic partner who embraces *familismo* values (β = 0.49), which, in turn, predicted their intentions to have sex (β = −0.19), adolescent acculturation did not significant predict *familismo*. Males: Higher acculturation was associated with a lowered preference for romantic partners who embrace *familismo* (β = −0.23), which, in turn, were associated with stronger intentions to engage in sex in the near future (β = −0.15). As the mother became more acculturated, her embracement of *familismo* weakened (β = −0.17) which, in turn, was associated with greater acculturation on the part of the boy (β = −0.35). This, in turn, translated into stronger intentions to have sex (β = −0.15).
Minnis et al., (2010) [76]	Familism	Sexual partner’s risk characteristics: (1) had concurrent partners; (2) used alcohol and/or marijuana at least weekly; and (3) belonged to a gang or was incarcerated during their sexual relationship.	Prospective longitudinal cohort study. *n* = 411 (55.4% females) *M* age = 16.6 Country of origin not detailed.	Immigration generation (recent immigrant; 1.5 generation; second; and third generation).	Familism did not mediate the relationship between immigrant generation and either of the two partner risks for which significant differences by generation were found (i.e., partner used alcohol/marijuana frequently OR = 1.0; and partner gang affiliated/incarcerated OR = 1.1).
Schwartz et al. (2014) [88]	Cultural values of individualism and collectivism.	Number of oral sex and vaginal/anal sex partners, and unprotected sex.	Prospective longitudinal design. *n* = 302 (53 % boys) *M* age = 14.51 Recently immigrated primarily from Mexico (35%) and Cuba (31%).	Years in the U.S. (covariate).	For males, individualist values were risk-enhancing via number of oral sex partners (OR = 4.02), and collectivist values were associated with fewer sexual partners (OR = 0.57). These findings were significant when controlling for migration status in terms of years in the U.S.
Velasquez et al., (2017) [85]	*Familismo*	Condom-use attitudes and intentions to use condoms.	Cross-sectional study. *N* = 128 (56.3% female) *Mean age* = 15.30 years, SD = 1.61. Country of origin not detailed.	ARSMA-II ^f^.	Adolescents who endorsed high levels of *familismo* (β = 0.31, *p* = 0.002) reported more favorable condom-use attitudes whereas adolescents who endorsed high traditional gender roles (β = –0.24, *p* = 0.01) held less favorable attitudes. Acculturation did not (β = 0.02, *p* = 0.83) predict attitudes. In addition, traditional gender roles (β = 0.37, *p* = 0.000) significantly predicted condom-use intentions, yet *familismo* (β = −0.15, *p* = 0.12) and acculturation (β = 0.10, *p* = 0.36) did not.
Religiosity					
Adam et al., (2005) [89]	Religiosity	Onset of sexual intercourse.	Cross-sectional design. *N* = 3187 (55% females) *M* age = 14.68. Country of origin not detailed.	Primary language spoken.	Religiosity was significantly related to onset of sexual intercourse (OR = 0.46). The risk for experiencing onset of intercourse was amplified for highly acculturated Hispanic adolescents (OR = 1.69). However, less acculturated Hispanic youth were actually less likely to have experienced first intercourse than English-speaking Hispanic youth (OR = 0.35), or bilingual Hispanic youth (OR = 0.45).
Edwards et al., (2008) [90]	Religiosity including traditional attitudes on sexuality.	Ever being sexually active, number of sexual partners, and age of sexual debut.	Cross-sectional study. *N* = 570 (49.65% females) Mean age was 17.91 years Country of origin not detailed.	Interview language preference (assimilated = English preference; unassimilated = Spanish preference).	Unassimilated adolescents who held religion as very important, attended church at least once a week, and had traditional attitudes on sexuality were less likely to ever have had sex, more likely to have had fewer sexual partners in the past 12 months (F = 5.21, 5.62, 6.73, religiosity predictors, respectively) and in their lifetime (F = 5.96, 6.42, 5.54, religiosity predictors, respectively), and to have had a later age at coital debut (F = 4.89, 9.20, 14.96 religiosity predictors, respectively).
Ertl et al., (2018) [91]	Positive religious coping	Condom use, number of sexual partners, and sex under the influence of alcohol	Cross-sectional data. *n* = 530 Latina women *M* age = 20.81, *SD* = 1.82. Country of origin not detailed.	Time in the U.S. and the SMAS ^i^.	Number of sexual partners was positively related to time in the US (r = 0. 33) but was negatively associated with positive religious coping (r = −0.16). Sex under the influence of alcohol was positively associated with time in the US (r = 0.10) but was not related to religious coping.
Guilamo-Ramos et al., (2005) [92]	Religiosity and religious affiliation	Virgin status, vaginal intercourse, birth control/condom use at most recent intercourse, and pregnancy.	Prospective longitudinal study (Add Health; 1994–1996). *n* = 2035 Med. Age = 15. Country of origin not detailed.	Language spoken and length of time in the U.S.	The predicted odds of being a non-virgin are somewhat higher for Spanish speakers as opposed to English speakers when the number of years living in the United States is low (OR = 0.24). There were no moderating effects of religion or religiosity.
Nadeem, Romo, and Sigman (2006) [77]	Religion and religiosity (Catholic vs. non-Catholic)	Knowledge about condom use.	Cross-sectional study. *n* = 45 unmarried pregnant Latina female adolescents *M* age = 16.5 Country of origin not detailed.	Language (Spanish vs. English).	No link was found between maternal communication and language, religiosity, and religion (Catholic vs. non-Catholic).
Raffaelli, Zamboanga, and Carlo (2005) [93]	Religiousness	Sexual behaviors (voluntary and involuntary)	Cross-sectional study. *n* =61 females *M* age = 18.4 Cuban origin or descent.	Nativity, childhood and current language, and ethnic identity (using the MEIM ^h^).	Less religiousness (β = −0.46), being U.S. born (β = −0.34), and more ethnically identified (β = 0.36) were individual predictors of higher sexual risk. Acculturation measures did not significantly predict sexual activity while religiousness (β = 0.25) remained a significant predictor.
Smith (2015) [94]	Intrinsic and extrinsic religiosity.	Early sexual debut; number of sex partners; condom use; sex with an intravenous drug user; tested for STIs; and sex under the Influence of alcohol/drugs.	Cross-sectional design. *n* = 1168 females *M* age = 15.81 Country of origin not detailed.	Linguistic acculturation (language spoken at home and language used for survey).	Latina females with lower levels of acculturation (β = 0.37), higher levels of intrinsic religiosity (β = 0.35) are less likely to engage in risky sexual behavior. Extrinsic religiosity (β = 0.24) functioned as a risk factor, increasing the likelihood of engaging in risky sexual behavior.
Traditional Gender Norms					
Deardorff et al., (2010) [58]	*Marianismo* (importance of female virginity). *Machismo* (importance placed of satisfying sexual needs).	Age at sex initiation (vaginal and anal), number of sexual partners and frequency of condom use	Cross-sectional design. *n* = 839 (55% females) *Mean* age = 18.5 Country of origin not detailed.	Language use (BAS ^a^).	Among females, importance attached to female virginity (marianismo) was negatively associated with lifetime number of sexual partners (OR = 0.8) and in the past year (OR = 0.9), and was positively associated with non-use of condoms (OR = 1.8). In addition, considering satisfaction of sexual needs important (machismo) was associated with more sexual partners among those who attached little value to female virginity (OR = 0.7). For males, the importance of satisfying sexual needs increased with the number of lifetime (OR = 1.4), recent sexual partners (OR = 1.1), and with inconsistent condom use (OR = 1.9).
Deardorff et al., (2013) [95]	Sexual values (e.g., considering sexual talk disrespectful and female virginity important)	Condom negotiation strategies to engender or avoid condom use.	Cross-sectional data collected *n* = 571 (61% females) *Mean* age = 18.44 Country of origin not detailed.	Nativity and linguistic acculturation (assessed using subscale of the BAS ^a^).	Among females, comfort with sexual communication (β = 0.32) was positively associated with frequency of communication to foster condom use. Premarital virginity importance (β = 0.61) was positively associated with expressing dislike of condoms. Moreover, the degrees to which women considered sexual talk disrespectful (β = 0.48) and female virginity important (β = 0.42) were positively associated with shared risk information as a condom-use strategy. Among both sexes, importance placed on premarital female virginity was negatively associated with use of direct communication strategies (β = −0.12).
Ertl et al., (2018) [91]	*Mariansimo* (pillar of family and chastity)	Condom use, number of sexual partners, and sex under the influence of alcohol	Cross-sectional data. *n* = 530 Latina women *M* age = 20.81, *SD* = 1.82. Country of origin not detailed.	Time in the U.S. and the Stephenson Multigroup Acculturation Scale (SMAS) ^i^ assessed changes in cultural practices.	Number of sexual partners was positively related to time in the US (r = 0. 33) and endorsing the family pillar belief (r = 0.09). Factors negatively associated with number of sexual partners included endorsing the virtuous and chaste belief (r = −0.21) and the subordinate/self-silencing belief (r = −0.32). Sex under the influence of alcohol was positively associated with time in the US (r = 0.10), and, conversely, was negatively related to endorsing the virtuous and chaste belief (r = −0.11).
Espinosa-Hernández et al., (2013) [86]	Importance of female virginity.	Timing of sexual behaviors (making out, receiving and performing oral sex, and vaginal sex).	Retrospective study. *n* = 153 female adolescents *M* age = 16.3 Mexican origins	Nativity and language use measured by the BAS ^a^.	Participants who were born in the U.S. were less likely to have engaged in making out (HR = 1.82) when accounting for the importance placed on female virginity. For all behaviors, placing a greater value on female virginity was associated with lesser odds of engaging in that behavior (making out HR = 0.77; performing oral sex HR = 0.68; receiving oral sex HR = 0.89; vaginal sex HR = 0.69).
Kaplan, Erickson, and Juarez-Reyes (2002) [61]	Gender role orientation	Age at first intercourse, number of lifetime sexual partners, and number of pregnancies.	Cross-sectional study. *n* = 670 Latina female adolescents *Mean* age = 17.5 Country of origin not detailed.	Linguistic acculturation (preferred language for speaking, reading, and writing).	Lower acculturation level (β = −0.19) and higher traditional the gender role orientation (β = –0.13) was related to older age of first intercourse. Once sexually active, acculturation and substance experimentation become more important influences on sexual risk-taking among Latina adolescents.
Velazquez et al., (2017) [85]	Traditional gender roles *Familismo.*	Condom-use attitudes and intentions to use condoms in the future.	Cross-sectional study. *N* = 128 (56.3% female) *Mean age* = 15.30 years, SD = 1.61. Country of origin not detailed.	ARSMA-II ^f^	Cultural values of *familismo* (β = 0.31, *p* = 0.002) and traditional gender roles (β = −0.24, *p* = 0.01) significantly predicted condom-use attitudes, while acculturation did not (β = 0.02, *p* = 0.83). In addition, traditional gender roles (β = 0.37, *p* = 0.000) significantly predicted condom-use intentions, yet *familismo* (β = −0.15, *p* = 0.12) and acculturation (β = 0.10, *p* = 0.36) did not.

Note: Ten studies are included in more than one section of the table because they examined more than one social and/or cultural factor. * OR = Odds ratio; HR = Hazards ratio; RRR = Relative risk ratio. ^a^ Bidimensional Acculturation Scale [13]. ^b^ Bicultural Involvement Questionnaire-Short Version (BIQ-S) [96]. ^c^ Bicultural Involvement Questionnaire-Revised [97]. ^d^ Short Acculturation Scale for Hispanics [98]. ^e^ Short Acculturation Scale for Hispanic Youth [99]. ^f^ Acculturation rating scale for Mexican Americans -II [14]. ^g^ Bicultural Involvement Questionnaire [100]. ^h^ Multi-Ethnic Identity Measure [101]. ^i^ Stephenson Multigroup Acculturation Scale (SMAS) [102].

**Table 2:** Summary of acculturation scales used in included studies.

Acculturation Scale	# of Items (Subscales)	Description
^a^ Bidimensional Acculturation Scale [13].	24 (2)	Measures two major dimensions of acculturation (Hispanic and non-Hispanic) using 12 items (per cultural domain) measuring 3 language-related areas (English media use, language use, and proficiency).
^b^ Bicultural Involvement Questionnaire-Short Version [96]	24 (2)	Consists of 12 items assessing U.S. practices (e.g., speaking English, eating U.S. foods) and 12 items assessing Hispanic practices (e.g., speaking Spanish, eating Hispanic foods).
^c^ Bicultural Involvement Questionnaire-Revised [97].	42 (2)	Measures the level of orientation toward American (21 items) and Hispanic (21 items) cultures in terms of both (a) comfort with and enjoyment of American and Hispanic cultural practices (e.g., comfort and use of language, food, and traditions) and (b) how much participants would want or like to utilize American and Hispanic cultural practices.
^d^ Short Acculturation Scale for Hispanics [98].	12 (3)	Measures 3 aspects of acculturation: (a) language use (5 items), (b) media use (3), and (c) ethnic social relations (4 items).
^e^ Short Acculturation Scale for Hispanic Youth [99].	12 (3)	Measures 3 dimensions of acculturation in Hispanic youth: (a) extrafamilial language use (3 items), (b) familial language use (6 items), and (c) ethnic social relations (2 items).
^f^ Acculturation rating scale for Mexican Americans -II (ARSMA-II) [14].	30 (2)	Measures behavioral and affective aspects of acculturation. Two subscales (measuring integration and assimilation, as well as marginalization and separation) capturing orientations to Anglo (13 items) and Mexican (17 items) culture independently. Assesses four domains: (a) language use and preference, (b) ethnic identity and classification, (c) cultural heritage and ethnic behaviors, and (d) ethnic interaction.
^g^ Bicultural Involvement Questionnaire [100].	42 (21)	Measures language use and involvement in both Latino and mainstream American activities. It yields two sets of scores to derive a measure of bicultural involvement, with individuals who are highly involved in both cultures scoring highest on the scale. A total of 21 items assess U.S. practices (e.g., speaking English, eating American food, associating with American friends), and the other 21 items assess Hispanic practices (e.g., speaking Spanish, eating Hispanic food, associating with Hispanic friends).
^h^ Multi-Ethnic Identity Measure [101].	15 (2)	Measures ethnic identity based on the elements that are common across groups so that it can be used with all ethnic groups. Comprised of 2 factors, ethnic identity search (5 items; a developmental and cognitive component) and affirmation, belonging, and commitment (7 items; an affective component). Three items are used only for the purposes of identification and categorization by ethnicity.
^i^ Stephenson Multigroup Acculturation Scale (SMAS) [102].	32 (2)	Measures the strength of practices in one’s heritage culture and U.S. culture. Created to measure engagement in cultural practices among members of any ethnic group and not one specific group. Exploratory factor analyses generated a 2-factor solution from the 32-item questionnaire: ethnic society immersion and dominant society immersion.

Note: Superscript notation correspond to notation of position in Table 1.

## Data Availability

No new data were created or analyzed in this study. Data sharing is not applicable to this article.
